# Performance Differences Using a Vibro-Tactile P300 BCI in LIS-Patients Diagnosed With Stroke and ALS

**DOI:** 10.3389/fnins.2018.00514

**Published:** 2018-07-31

**Authors:** Alexander Heilinger, Rupert Ortner, Vincenzo La Bella, Zulay R. Lugo, Camille Chatelle, Steven Laureys, Rossella Spataro, Christoph Guger

**Affiliations:** ^1^g.tec medical engineering GmbH, Schiedlberg, Austria; ^2^g.tec medical engineering Spain SL, Barcelona, Spain; ^3^ALS Clinical Research Center, BioNeC, University of Palermo, Palermo, Italy; ^4^GIGA Consciousness, Coma Science Group, University of Liège, Liège, Belgium; ^5^French Association of Locked-in Syndrome (ALIS), Paris, France; ^6^Research Department, Hospital Universitari Institut Pere Mata, Reus, Spain; ^7^Centro Neurolesi Bonino Pulejo (IRCCS), Palermo, Italy; ^8^Guger Technologies OG, Graz, Austria

**Keywords:** locked-in syndrome, BCI performance, stroke, ALS, tactile stimulation, P300 event-related potential

## Abstract

Patients with locked-in syndrome (LIS) are typically unable to move or communicate and can be misdiagnosed as patients with disorders of consciousness (DOC). Behavioral assessment scales are limited in their ability to detect signs of consciousness in this population. Recent research has shown that brain-computer interface (BCI) technology could supplement behavioral scales and allows to establish communication with these severely disabled patients. In this study, we compared the vibro-tactile P300 based BCI performance in two groups of patients with LIS of different etiologies: stroke (*n* = 6) and amyotrophic lateral sclerosis (ALS) (*n* = 9). Two vibro-tactile paradigms were administered to the patients to assess conscious function and command following. The first paradigm is called vibrotactile evoked potentials (EPs) with two tactors (VT2), where two stimulators were placed on the patient’s left and right wrist, respectively. The patients were asked to count the rare stimuli presented to one wrist to elicit a P300 complex to target stimuli only. In the second paradigm, namely vibrotactile EPs with three tactors (VT3), two stimulators were placed on the wrists as done in VT2, and one additional stimulator was placed on his/her back. The task was to count the rare stimuli presented to one wrist, to elicit the event-related potentials (ERPs). The VT3 paradigm could also be used for communication. For this purpose, the patient had to count the stimuli presented to the left hand to answer “yes” and to count the stimuli presented to the right hand to answer “no.” All patients except one performed above chance level in at least one run in the VT2 paradigm. In the VT3 paradigm, all 6 stroke patients and 8/9 ALS patients showed at least one run above chance. Overall, patients achieved higher accuracies in VT2 than VT3. LIS patients due to ALS exhibited higher accuracies that LIS patients due to stroke, in both the VT2 and VT3 paradigms. These initial data suggest that controlling this type of BCI requires specific cognitive abilities that may be impaired in certain sub-groups of severely motor-impaired patients. Future studies on a larger cohort of patients are needed to better identify and understand the underlying cortical mechanisms of these differences.

## Introduction

The term locked-in syndrome (LIS) was introduced to describe a clinical state of quadriplegia and anarthria due to a disruption of the corticospinal and corticobulbar tracts in the brainstem (Plum and Posner, 1983). The principal etiology of acute onset LIS is stroke (ischemic or hemorrhagic) affecting the ventral part of the pons (Patterson and Grabois, 1986). LIS can also result from the late stage of chronic degenerative neurological diseases such as amyotrophic lateral sclerosis (ALS), which affects the upper and lower motor neurons, leading to progressive paralysis of voluntary muscles and eventually to respiratory failure (Bäumer et al., 2014).

Based on the severity of motor deficits, three varieties of LIS have been described: classical LIS, in which the patient is unable to move – except for eye movements or blinking – or to speak; incomplete LIS, in which residual voluntary movements in addition to eye movements can be present; and total or complete LIS (CLIS), where patients show total immobility, including lack of voluntary eye movement (Bauer et al., 1979). Patients with CLIS/LIS can be mistaken with patients in coma or with other DOC such as the vegetative state/unresponsive wakefulness syndrome (VS/UWS), in which patients are eyes opened but do not show any sign of voluntary movement. Hence, reliable diagnostic tools for the differentiation of these clinical conditions are of utmost importance.

Despite the existence of well-defined clinical criteria for the diagnosis of DOC and LIS, differential diagnosis remains challenging and misdiagnosis still occurs. Standardized behavioral scales like the Glasgow Coma-Scale (GCS) (Teasdale and Jennett, 1974) and the Coma-Recovery-Scale revised (CRS-R) (Giacino et al., 2004) are widely used in clinical settings. However, such tools are limited when assessing patients with CLIS as they are highly dependent on motor abilities. For these patients, supplementary tools are needed.

Once the diagnosis of LIS has been established, another major challenge with this population is providing them with appropriate devices for communication and environmental management. These tools can increase quality of life and facilitate the assessment of cognitive impairments (e.g., fronto-temporal dementia), which has been described to be often associated with ALS (Phukan et al., 2007).

In this context, brain-computer interface (BCI) systems have been used for decades to establish communication with patients with LIS, usually via the electroencephalogram (EEG) (Wolpaw et al., 2002; Wolpaw and Wolpaw, 2012). Different EEG paradigms have been employed that use different stimuli or mental tasks, including motor imagery, steady-state visual evoked potentials (EPs) (Guger et al., 2003; Bin et al., 2009; Combaz et al., 2013; Ahn et al., 2015) and event-related potentials (ERPs), notably the P300 waveform (Fazel-Rezai et al., 2012; Blankertz et al., 2016). Most P300-BCIs rely on the visual modality, but auditory or vibro-tactile modalities have been explored for patients with visual/auditory impairments, which have been described to be present in an important percentage of patients with LIS (Lugo et al., 2015).

Kaufmann et al. (2013) compared different BCI modalities on a single LIS patient, reporting that the tactile modality was clearly superior compared to visual or auditory modalities. Prior work has shown that healthy subjects without prior training could achieve a mean classification accuracy of 93% with a vibro-tactile paradigm (Alison et al., 2017). Using the same method, 12 ALS patients (9 LIS/3 CLIS) achieved a median accuracy of 76.6% (min: 40/max: 100) using a vibro-tactile paradigm with two stimulators (VT2) (Guger et al., 2017). The same publication showed that 2/3 CLIS patients reached a classification of 100% using VT3. These two CLIS patients could also communicate correctly (9/10 and 8/10 questions answered correctly). In other work using vibrotactile P300 BCI for LIS patients, six patients achieved an average accuracy of 80% (min: 20%/max: 100%) in a paradigm with VT2 and 55.3% (min: 20%/max: 100%) in a paradigm with VT3 (Lugo et al., 2014).

Silvoni et al. (2016) investigated the neurophysiological correlates of vibrotactile stimulation processing in a group of 14 ALS patients and 10 healthy subjects, using a single vibro-stimulator placed on the left hand. They reported that responses to tactile stimuli were not altered in ALS, suggesting that this neurophysiological signal could be used in at least some ALS patients to control such a BCI.

In the current study, we investigated BCI performance in patients with LIS from different etiologies. We explored differences in classification accuracy and EPs using a vibro-tactile based BCI in two sub-groups of LIS due to ALS and stroke. Based on the literature suggesting preserved cognitive abilities in LIS patients from both etiologies (Phukan et al., 2011), we hypothesized that both groups would perform equally well using a vibro-tactile based BCI, even though the underlying pathological mechanisms differ between these two patient groups.

The results of this study could help to improve the assessment to detect the presence of consciousness in patients with stroke, ALS and other conditions. These findings may also help to shed light on the differences and clinical characteristics that should be considered with each patient group and underline the importance of a multimodal approach – using stimuli from different sensory modalities – to evaluate non-responsive patients.

## Materials and Methods

### Population

This retrospective study included data acquired in LIS patients at the University of Palermo, Italy (PA) and by the French Association of Locked-In Syndrome (ALIS) in Paris, France, as part of other studies previously published (Lugo et al., 2014; Guger et al., 2017). For the ALS patients, the following inclusion criteria were used: patients had to be over 18 years old, diagnosed with definite ALS according to the El Escorial Diagnostic Criteria and LIS/CLIS state verified by experienced neurologists in motor neuron diseases, without evidence of cognitive and behavioral abnormalities along the disease’s course. For stroke patients, the following inclusion criteria were used: the patients had to be over 18 years old and diagnosed with stroke in the chronic (>1 year since diagnosis) LIS state.

**Table 1** reports the patients’ demographic data. We included a convenience sample of six stroke patients (three ischemic, three hemorrhagic; median age = 40, min: 21, max: 48) with a disease duration between 4 and 19 years (median = 10), and nine ALS patients (median age = 59, min: 37, max: 68) with a disease duration between 2, 3, and 12 years (median = 7). The difference between gender and age was tested using a Chi-Square-Test (significance = *p* < 0.05). There was no difference in age and gender between the two groups.

**Table 1:** Overview of all patients including demographic information, classification accuracies, and communication mode results.

Patient	Sex	Age	Diagnosis	Disease duration (years)	Clinical syndrome	Paradigm	Accuracy [%]	Communication using VT3
S1	F	47	Stroke (ischemic)	4	LIS	VT2-1	79.9	4C/6U
						VT2-2	45.6	
						VT3-1	52.8	
						VT3-2	23.1	
S2	F	21	Stroke (ischemic)	4	LIS	VT2-1	42.2	10U
						VT2-2	16.5	
						VT3-1	28.3	
						VT3-2	13.4	
S3	M	46	Stroke (ischemic)	16	LIS	VT2-1	13.6	2C/8U
						VT2-2	10.1	
						VT3-1	35.5	
						VT3-2	7.9	
S4	M	33	Stroke (hemorrhagic)	12	LIS	VT2-1	44.4	6C/4U
						VT2-2	40.6	
						VT3-1	30.5	
						VT3-2	21.45	
S5	F	48	Stroke (hemorrhagic)	5	LIS	VT2-1	61.1	2C/8U
						VT2-2	9.0	
						VT3-1	14.2	
						VT3-2	14.2	
S6	F	46	Stroke (ischemic)	19	LIS	VT2-1	25.0	10U
						VT2-2	22.9	
						VT3-1	24.5	
						VT3-2	19.0	
A1	F	68	ALS	7.5	LIS	VT2	100.0	9C/1W
						VT3	99.6	
A2	F	65	ALS	7	LIS	VT2	21.6	10U
						VT3-1	12.4	
						VT3-2	1.6	
A3	F	65	ALS	7	LIS	VT2	100.0	7C/1W/2U
						VT3	90.0	
A4	F	76	ALS	12	LIS	VT2	100.0	8C/1U/1W
						VT3	87.5	
A5	F	46	ALS	11.3	LIS	VT2	98.2	7C/3U
						VT3-1	81.4	
						VT3-2	51.5	
A6	M	63	ALS	2.3	LIS	VT2	97.2	9C/1W
						VT3	91.0	
A7	M	68	ALS	4.3	LIS	VT2	94.2	8C/2W
						VT3	100.0	
A8	M	37	ALS	8.5	LIS	VT2	100.0	8C/2W
						VT3-1	52.4	
						VT3-2	95.1	
A9	F	47	ALS	2.5	LIS	VT2	21.2	7C/2U/1W
						VT3	99.7	

*F, female; M, male; C, correct answers; U, undecided answers; W, wrong answers. Each participant completed one communication run with 10 questions*.

### Brain-Computer Interface System

The mindBEAGLE system (g.tec Guger Technologies OG, Austria) was used for all data collection and real-time feedback. The system uses active gel-based EEG electrodes connected to a biosignal amplifier (g.USBamp, g.tec medical engineering GmbH). The amplifier has a 24-bit resolution and a high oversampling rate to increase the signal-to-noise ratio. The amplifier is connected to the computer via USB and sends the data in real-time at a sampling rate of 256 Hz. The EEG signal is presented on a monitor for quality inspection during the measurement, and the data are stored in floating point format for later data analysis.

The recorded EEG data were filtered between 48 and 50 Hz using a notch filter. Afterward the data were bandpass filtered between 0.1 and 30 Hz to remove baseline shifts and eliminate most of the EEG artifacts. Eight electrodes were used for the recording, placed on the Fz, C3, Cz, C4, CP1, CPz, CP2, and Pz position according to the extended international 10–20 electrode system. The reference electrode was fixed on the right earlobe and the ground electrode was mounted on the forehead.

### Paradigm

Two P300 oddball paradigms were used: vibrotactile EPs with two tactors (VT2) and vibrotactile EPs with three tactors (VT3). Both paradigms presented 480 stimuli per run, with 60 groups of 8 stimuli. In both paradigms, the patient was instructed via earbuds to silently count vibrotactile pulses to either the left or right wrist. The left and right wrists had an equal chance of being chosen pseudo-randomly as the “target” wrist. All vibrotactile stimuli lasted 100 ms, with a 100 ms delay between stimuli. Both paradigms required about 2.5 min per run and were designed to elicit an oddball P300 to stimuli delivered to the target wrist only.

In the VT2 paradigm, the two tactors were placed on the left and right wrists. Each of the 60 groups of eight stimuli per run contained one target and seven non-target stimuli, presented in pseudorandom order. Thus, the target to non-target ratio was 1:7.

In the VT3 paradigm, an additional (third) tactor was placed on a third location on the patient’s body. For the ALS patients, the third tactor was placed on the upper part of the back. For the stroke patients, the third tactor was placed on the neck. The position of the third tactor can be arbitrary, since it acts as a distractor. The other two tactors were fixed on the right and left wrists. In VT3, each sequence of eight stimuli included one stimulus to the left wrist, one stimulus to the right wrist, and six stimuli to the third tactor, in pseudorandom order. Thus, each sequence of eight stimuli also contained one target, like the VT2 paradigm, but six of the seven non-targets were meant as “distractor” stimuli that could never be designated as the target. The runtime for both VT modes was 2.5 min for one run.

In addition to these two paradigms to assess patients, we also explored communication using the VT3 paradigm. The experimenter asked yes/no questions and the patient was asked to answer by counting the stimuli on either the left or right wrist. One question can be answered after 120 stimuli, which requires 38 s. The system only selects YES or NO if the result is significant and presents no response otherwise. This result is presented to the experimenter via the monitor. Each patient was asked 10 questions. The communication was considered reliable if the patient could accurately answer at least 7/10 questions.

Each patient participated in one experimental session that included one VT3 communication run and at least one run (each) of VT2 and VT3 assessment. Some patients participated in additional VT2 and/or VT3 runs, as shown in **Table 1**. The total number of runs per session was limited to five per participant, to address concerns with possible fatigue or discomfort.

### Data Analysis

For both paradigms, data segments of -100–600 ms around each stimulus were extracted. Each of these single trials was baseline corrected and averaged. Trials in which the EEG signal amplitude exceeded ±100 μV were rejected from the EP and classifier calculation. The EPs were visually inspected. For the classification, the data were classified using the linear discriminant analysis (LDA), which resulted in a classification accuracy between 0 and 100%. This result showed how well the data could be discriminated using the classifier. In one assessment run, 60 sequences of tactile stimuli were presented to the patient. Each sequence contained eight trials in randomized order, in which one trial was the target trial and seven were non-target trials. This resulted in 480 trials total (60 target trials/420 non-target trials). After removal of artifact trials, the ratio between non-target and target trials was set to 7:1 again. Trials in both pools were shuffled and split 50:50 into training and testing data. The training data were used to create an LDA classifier and tested on the testing data as follows: seven non-target trials and one target trial were randomly chosen and the LDA score was calculated for each of the trials. The trial with the highest LDA score was classified as target trial. If the classified target trial was the real target trial, the test classification was correct (100%), otherwise incorrect (0%). This step was repeated 1000 times, resulting in a classification plot.

Brain-computer interface performance was considered above chance when the classification accuracy was higher than 23% based on binomial distribution (Ortner et al., 2014). Non-parametric statistical method was chosen due to the small sample size. To compare the classification accuracies between the two groups, a Wilcoxon rank sum test was used. VT2 and VT3 paradigm results were compared separately. We considered significance at *p* < 0.05.

## Results

### Classification Accuracy

**FIGURE 1:**
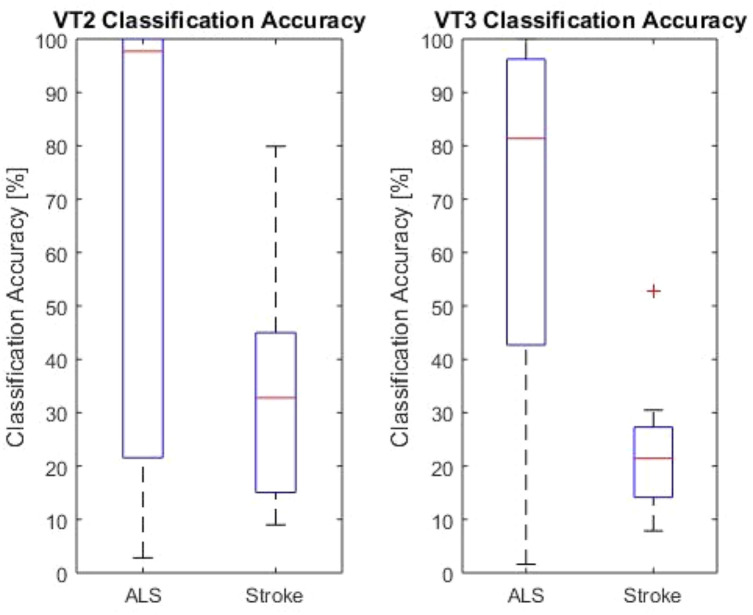
VT2 and VT3 classification accuracies for both LIS groups. The red lines indicate the mean values for the group. The upper and lower end of the box represents the first quartile. The black lines are the end of the first and fourth quartile. The red cross marks an outlier.

All patients performed the VT2 and VT3 paradigms, either once or multiple times. **Table 1** summarizes data obtained from all patients. All patients except one showed at least one run above chance in the VT2 paradigm. In the VT3 paradigm (i.e., active task), all 6 stroke patients and 8/9 ALS patients showed at least one run above chance. The ninth ALS patient (patient A2) attained at best 12.4% accuracy. Overall, patients achieved higher accuracies in VT2 than VT3. The accuracy observed during VT2 was higher in ALS patients than in stroke patients, with a median accuracy of 98% (min: 22%, max: 100%) and 32.8% (min: 15%, max: 45%), respectively (*p* < 0.05). For VT3, ALS patients also achieved a higher accuracy than stroke patients, with a median classification accuracy of 82% (min: 42%; max: 97%) and 22% (min: 15%; max: 28%), respectively (*p* < 0.01). The results are reported in **Figure 1**.

### Communication Mode

All patients participated in one communication run with 10 questions. None of the stroke patients could reliably communicate with the system. The classifier did not get any wrong answers for any of the stroke patients, but it did provide between 4 and 10 “undecided” answers.

For ALS patients, 8/10 patients could answer at least 7/10 questions accurately, while 2 were not able to reliably communicate with the system (the classifier provided “undecided” answers for all 10 questions).

### Evoked Potentials

**FIGURE 2:**
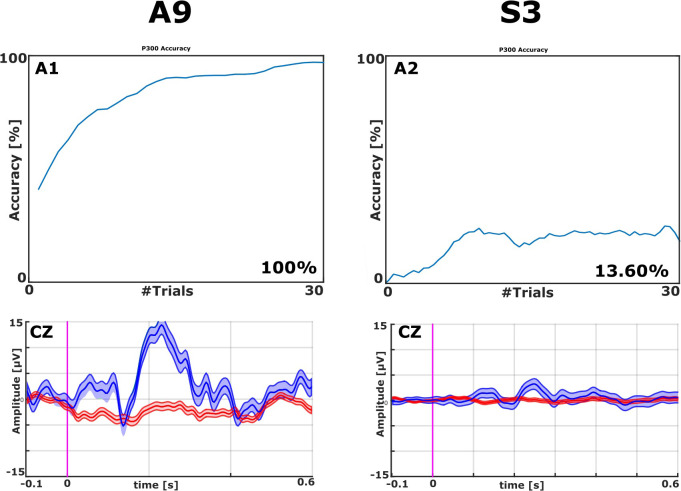
Examples of EPs and Classification Accuracies from ALS patient A9 and stroke patient S3, both from the VT2 paradigm. The classification accuracy plot on the top left shows that one ALS patient achieved 100% accuracy (top left). The classifier could effectively discriminate target from non-target stimuli after about 10 trials with A9’s data. Visual inspection of the averaged EPs (bottom left) shows a clear N1 followed by a robust P2 (Amplitude > 15 μV over site Cz) in patient A9 to target trials (blue lines). The non-target trials (red lines) did not exhibit these features. The top right panel shows that one stroke patient attained 13.6% accuracy. Concordantly, the ERPs in the bottom right do not exhibit robust differences between target and non-target ERPs.

All EPs were visually inspected. With the VT2 paradigm, all patients showed a high P300 complex. **Figure 2** presents examples with classification accuracy and the EPs on the Cz electrode from patients A9 and S3.

**FIGURE 3:**
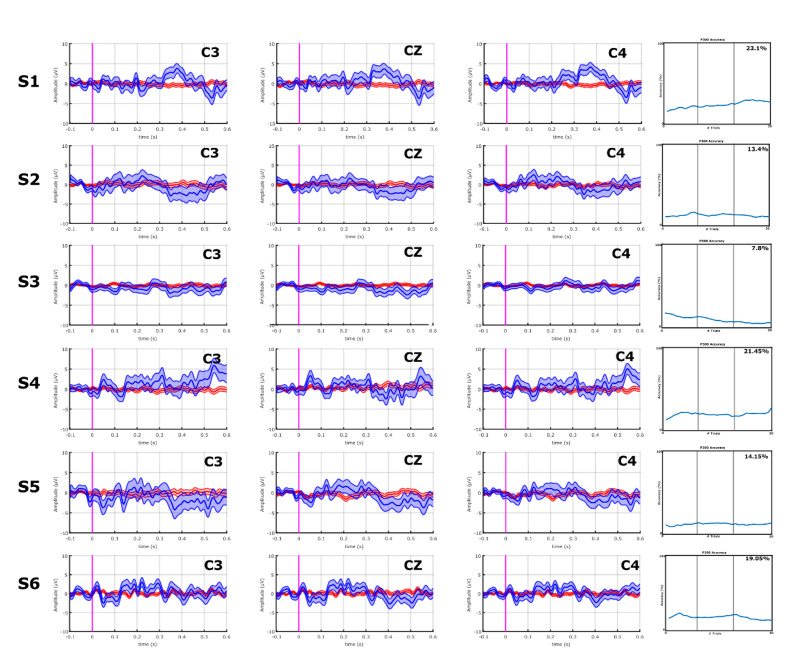
EPs and classification accuracies of all stroke patients from the VT3 communication runs. All stroke patients produced a P300 ≤ 5 μV, and a classification accuracy of 23.1% (patient S1) or lower in the VT3 runs. Visual inspection of **Figures 3** and **4** shows that the differences in the target vs. non-target EPs in the stroke patient group appear small compared to the ALS group, and the classifier could not find consistent and robust differences.

**FIGURE 4:**
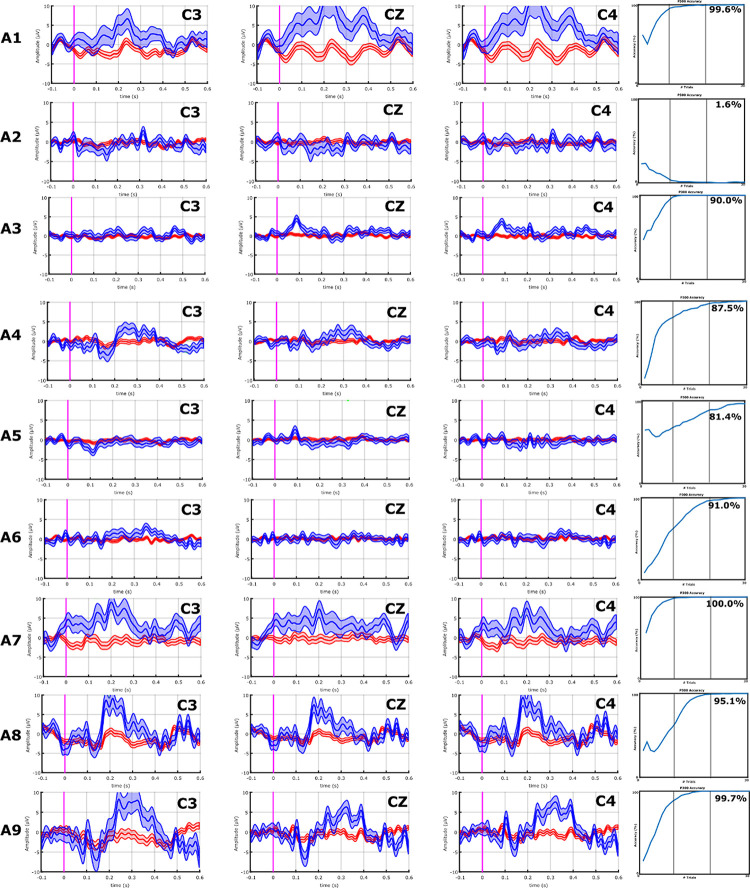
EPs and classification accuracies of all ALS patients from the VT3 communication runs. ALS patients A1, A8, A9, and A10 had a P2 and/or P3 greater than 5 μV over C3, Cz, and C4. Consistently, these ALS patients all achieved an accuracy ≥95%. In ALS patients A2–A7, the P300 response is lower than 5 μV or non-existent, and only A7 exhibits modest target vs. non-target differences in visual inspection. Nonetheless, the classifier found some activity to facilitate accurate classification, resulting in mean accuracies of 81.4% (patient A6) or higher, excluding patients A2 (VT3 = 1.6%) and A4 (VT3 = 16.3%).

With the VT3 paradigm, 4/10 ALS patients (A1, A7–A9) showed a high P300 or other components of the P300 complex, whereas none of the stroke patients did. These results can be seen in **Figures 3** and **4**.

## Discussion

The aim of this study was to compare performance with vibro-tactile BCI paradigms in patients with LIS resulting from ALS or stroke. Our hypothesis was that both groups would perform equally well using a vibro-tactile based BCI. Our results show higher performance in ALS than stroke patients, which might reflect the different pathological mechanisms underlying the LIS in each group.

The first explanation for the difference in both paradigms is the possible presence of reduced tactile sensitivity in patients with LIS due to stroke. Paterson and Grabois reported abnormalities of sensitivity in 34 of 62 patients (54%) (Patterson and Grabois, 1986) with LIS from various etiologies. This has also been reported in another study (Hawkes, 1974). Although it is not the main characteristic of the syndrome, the presence of such alterations is highly probable due to the possible lesion of the central lemniscus that runs just behind the pyramidal tracts.

We also observed lower accuracies in all patients during the consecutive runs except one in the ALS group. This could partly be explained by an increased fatiguability influenced by the lesion site of the stroke (Staub and Bogousslavsky, 2001). Even though the subject was asked to count the target, the responses to the VT2 paradigm could also be elicited without active participation of the person, which is not possible in VT3. This could also explain the decrease in performance. Additional research with more runs per patient, possibly across multiple sessions, could further elucidate the effects of consecutive runs.

Patient A9’s performance improved considerably between the two VT3 runs (VT3-1 = 52.4%; VT3-2 = 95.1%), which could be a short-term learning effect. The results from all other patients may reflect fatigue or an absence of learning effects, since the VT3 task entails a more challenging discrimination task. Further studies could explore whether this accuracy reduction correlates with mental exhaustion or other factors and develop new paradigms that might be less tiring. **Figure 4** show that the classifier could attain high accuracy with fewer than 30 groups of eight trials for most ALS patients, which suggests that shorter runs may be feasible.

During visual inspection of the EPs, all patients showed a P300 complex during VT2, but four ALS patients elicited a high P300 or other signal during VT3, whereas when none of the stroke patients did. If the high classification accuracies observed in the four ALS patients correspond with the EPs observed, the BCI system also found some additional components in the signal to produce high classification accuracy in the other patients. As VT3 is an active task, and therefore more cognitively demanding, the data suggest that there are some underlying cortical activities in ALS contributing to a high classification accuracy even if the EPs did not exhibit robust differences based on visual inspection of averaged data.

While the approach used here is often called a P300 BCI in the literature, many of the participants produced target vs. non-target differences in other ERP components of the P300 complex, notably the N100 and P200. Other articles have also noted that so-called P300 BCIs often rely on non-P300 components within the P300 complex (Fazel-Rezai et al., 2012; Allison et al., in review). Additional research is needed to further understand how these patients’ ERPs are generated and resulting clinical impact.

In summary, ALS patients showed high P300 amplitudes or other often atypical complexes, which could both be classified with high accuracies by the BCI system. Prior work has shown that LIS patients diagnosed with ALS could control a P300-based BCI system, sometimes over months (Sellers and Donchin, 2006; Nijboer et al., 2008; Silvoni, 2009). Comparing the EPs of the ALS group with the results of the stroke group, the most likely explanation for these differences is an alteration in tactile sensitivity.

Classification accuracy could indicate whether the patient will successfully communicate. Prior work showed that communication could be successful at a classification accuracy >60% (Guger et al., 2017). Within the stroke patient group, all answers that were not “correct” were “undecided.” As the stroke patients showed classification accuracies below 60% in the assessment runs, this could further suggest that the success of communication is dependent on classification accuracy. The ALS patient group showed a more heterogeneous response, with both “undecided” and “wrong” responses. This outcome could indicate that more stroke patients had concentration problems, fell asleep, forgot the instructions or were distracted. The possible presence of cognitive deficits in these patients must be also taken into account, since they have been described in previous studies of patients with LIS of vascular or traumatic etiology, especially with the presence of thalamic or hemispheric lesions (Schnakers et al., 2008; Rousseaux et al., 2009). In the case of patients with ALS, the A4 patient has associated a frontotemporal dementia, which may explain his poor performance and low response rate. The low classifier accuracy in the stroke patients and in ALS patients A2 and A4 might also accurately reflect that these patients are at least periodically unaware and/or unable to perform at least some of the mental tasks required for the paradigms used here.

This study used a montage with eight electrodes that were positioned to optimally record the P300. Previous work with a visual P300 BCI for spelling showed that a similar eight EEG electrode montage could yield a classification accuracy of 100% with 17 subjects (Guger et al., 2012). Additional electrodes did not substantially improve classifier accuracy (Guger et al., 2003). However, future work could explore expanded montages that could lead to better performance, especially with patient groups.

Several limitations have to be considered for this study. First, a small convenience sample of 15 LIS patients was included. We acknowledge the limitations of using a small sample size but emphasize the difficulty of measuring this specific patient population. Second, the threshold chosen for defining above chance level performance and communication runs might not be adapted for single patient performance, as it would require additional offline analyses. Finally, it would be interesting to document more extensively the cognitive abilities and the severity of disease of the patients. As this was a retrospective study, no further information could be extracted regarding each patient’s status, which also limits potential data analyses.

These results could contribute to improved mechanisms to assess the presence of consciousness in non-responsive patients, perhaps supplementing the CRS-R, GCS or other established clinical assessments. Our findings may also help to shed light on the differences and clinical characteristics that should be taken into account with each patient group and underline the importance of having a multimodal approach – using stimuli from different sensory modalities – to evaluate non-responsive patients to overcome sensory deficits and to adapt the means of communication to the remaining sensory capabilities.

The approach used here can objectively identify command following activity without any movement and could provide communication for some patients. All three parameters, i.e., classification accuracy, EPs and communication accuracy, might provide a more detailed and accurate information about each patient. However, results indicated that all three paradigms were more successfully used in ALS than stroke patients. As both the system and the paradigms used here are relatively new, substantial improvements are needed to answer these discrepancies.

## Summary

LIS Patients due to ALS attained better performance with the BCI paradigms employed in this study than LIS patients due to stroke. This could be explained by the lesion of the sensory pathways in patients with LIS due to brainstem stroke, and perhaps also a greater propensity toward fatigue in this group (such as due to brainstem damage). Future studies should elucidate these differences to design BCI paradigms that consider the underlying disease pathology, so as to best tailor BCIs accordingly for each patient.

## Author Contributions

ZL and RS were mainly responsible for data collection which occurred in collaboration with SL and VLB. AH was mainly responsible for data analysis and manuscript preparation. The remaining authors were responsible for study design and manuscript preparation.

## Conflict of Interest Statement

The authors declare that the hard- and software were made by g.tec medical engineering GmbH. Authors AH, RO, and CG are employed by g.tec. SL is on the scientific advisory board of g.tec. The remaining authors declare that the research was conducted in the absence of any commercial or financial relationships that could be construed as a potential conflict of interest.
