# Corrigendum: Performance Differences Using a Vibro-Tactile P300 BCI in LIS-Patients Diagnosed With Stroke and ALS

**DOI:** 10.3389/fnins.2020.637905

**Published:** 2021-01-06

**Authors:** Alexander Heilinger, Rupert Ortner, Vincenzo La Bella, Zulay R. Lugo, Camille Chatelle, Steven Laureys, Rossella Spataro, Christoph Guger

**Affiliations:** ^1^g.tec medical engineering GmbH, Schiedlberg, Austria; ^2^g.tec medical engineering Spain SL, Barcelona, Spain; ^3^ALS Clinical Research Center, BioNeC, University of Palermo, Palermo, Italy; ^4^GIGA Consciousness, Coma Science Group, University of Liège, Liège, Belgium; ^5^French Association of Locked-in Syndrome (ALIS), Paris, France; ^6^Research Department, Hospital Universitari Institut Pere Mata, Reus, Spain; ^7^Centro Neurolesi Bonino Pulejo (IRCCS), Palermo, Italy; ^8^Guger Technologies OG, Graz, Austria

**Keywords:** locked-in syndrome, BCI performance, stroke, ALS, tactile stimulation, P300 event-related potential

In the original article, we neglected to include the following funding information:

This project has received funding from the European Union's Horizon 2020 research and innovation programme under the Marie Skłodowska-Curie grant agreement No 712949 (TECNIOspring PLUS).

The authors apologize for this error and state that this does not change the scientific conclusions of the article in any way. The original article has been updated.

